# Secondary mid-term abdominal pregnancy: A case report

**DOI:** 10.1097/MD.0000000000043281

**Published:** 2025-07-18

**Authors:** Qi Wu, Suning Bai, Lina Han, Liyun Song

**Affiliations:** a Department of Obstetrics and Gynecology, Hebei General Hospital, Shi Jiazhuang, Hebei Province, China.

**Keywords:** abdominal pregnancy, case report, ectopic pregnancy, placenta

## Abstract

**Rationale:**

Ectopic pregnancy is a common acute abdominal disease of gynecology. Ectopic pregnancy refers to a pregnancy in which the fertilized egg is implanted outside the uterine cavity. Abdominal pregnancy is a type of ectopic pregnancy, which refers to the embryo or fetus being located in the abdominal cavity outside the fallopian tubes, ovaries, and broad ligaments. Abdominal pregnancy has a high misdiagnosis rate and mortality rate, seriously endangering maternal health. Therefore, early diagnosis and treatment can significantly improve patient prognosis.

**Patient concerns:**

A 34-year-old woman was admitted to our hospital with intermittent nausea and vomiting for 3 months.

**Diagnoses:**

The final diagnosis of this patient was abdominal pregnancy.

**Interventions:**

The patient underwent a cesarean exploration surgery, intraoperative ectopic pregnancy tissue clearance, partial omentectomy, left fallopian tube resection, and uterine fibroid resection.

**Outcomes:**

The patient recovered well 3 days after surgery and was allowed to be discharged. The patient was followed-up regularly.

**Lessons:**

The treatment of mid-pregnancy in the abdominal cavity includes conservative and surgical treatments. The key to successful treatment of mid-pregnancy in the abdominal cavity is the handling of the placenta. Currently, there is a lack of rich treatment experience in the industry for abdominal pregnancy in mid-to-late pregnancy, and there are different considerations for surgical and conservative treatment methods.

## 1. Introduction

Abdominal pregnancy means that the embryonic tissue is located in the abdominal cavity outside the fallopian tube, ovary, and broad ligament. With the improvement of living standards and the improvement of people’s healthcare awareness, the incidence rate of abdominal pregnancy is extremely low, about 1/25,000 to 1/10,000.^[1]^ The clinical manifestations of abdominal pregnancy are similar to those of tubal pregnancy, which are menopause with abdominal pain, occasional history of vaginal bleeding, abdominal pregnancy mass rupture, intraperitoneal bleeding, even shock, life threatening, and other risks. Therefore, once diagnosed, pregnancy should be terminated immediately.

## 2. Case report

**Figure 1.**
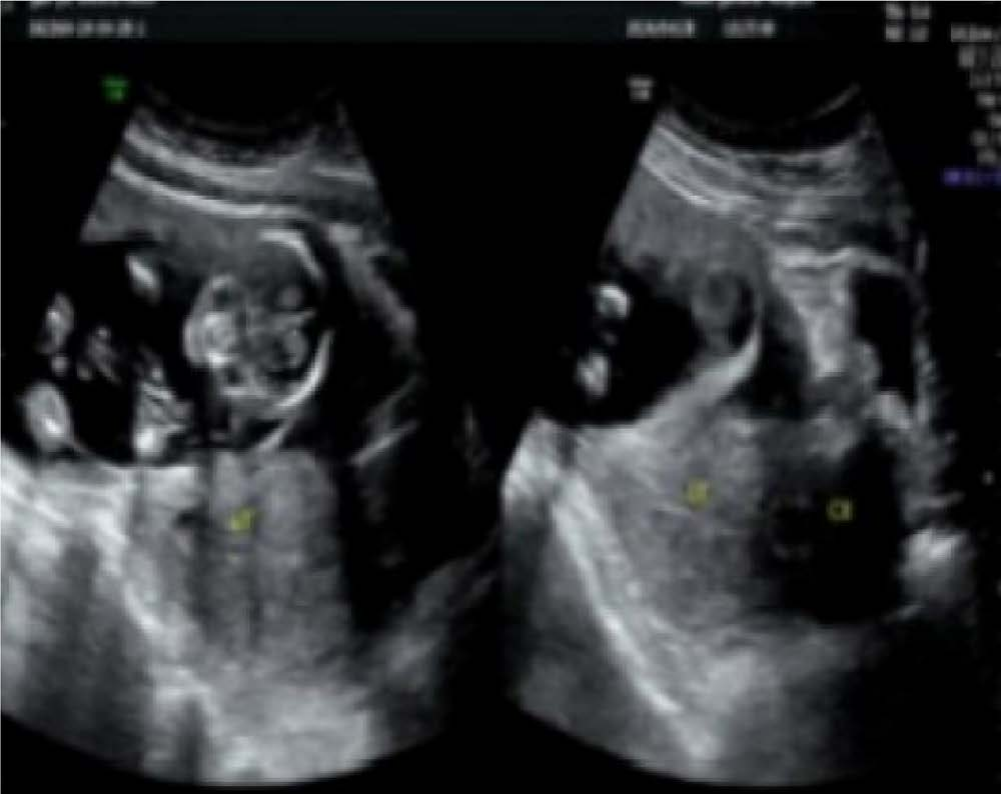
Abdominal pregnancy ultrasound image.

The patient is 34 years old, with 2 pregnancies and 1 delivery. The main cause is intermittent nausea and vomiting for 3 months. Abdominal pregnancy was discovered and admitted to our gynecology department on April 28, 2024. Three months ago, the patient had no obvious cause of nausea and vomiting, and self-tested negative for a urine pregnancy test. The patient received intravenous therapy at a local clinic for > 10 days (specific medication and dosage are unknown). The patient complained of regular vaginal bleeding every month, lasting for 2 days, with small amounts and occasional lower abdominal pain. The patient thought that their menstrual period was coming, and there was no discomfort, such as fever or diarrhea. Two days ago, the patient underwent a physical examination at Hebei International Travel Health Center, and a gynecological ultrasound showed that they were 3 months pregnant. The current patient requests diagnosis and treatment at our gynecology clinic. Gynecological ultrasound examination shows that the uterus is larger than normal, with an endometrial thickness of about 18 mm. A fetal sac can be seen in the anterior upper part of the uterus, and fetal imaging can be seen inside (biparietal diameter = 38 mm, head circumference = 134 mm, femur length = 20 mm, humerus length = 19 mm, fetal heart rate = 150 times/min); The depth of amniotic fluid is 43 mm, and the echo of the placenta can be seen behind the rectus abdominis muscle. Color Doppler flow imaging: rich vascular clusters can be seen on the outer side of the left lower edge of the placenta (Fig. 1). It is recommended to be hospitalized due to abdominal pregnancy.

**Figure 2.**
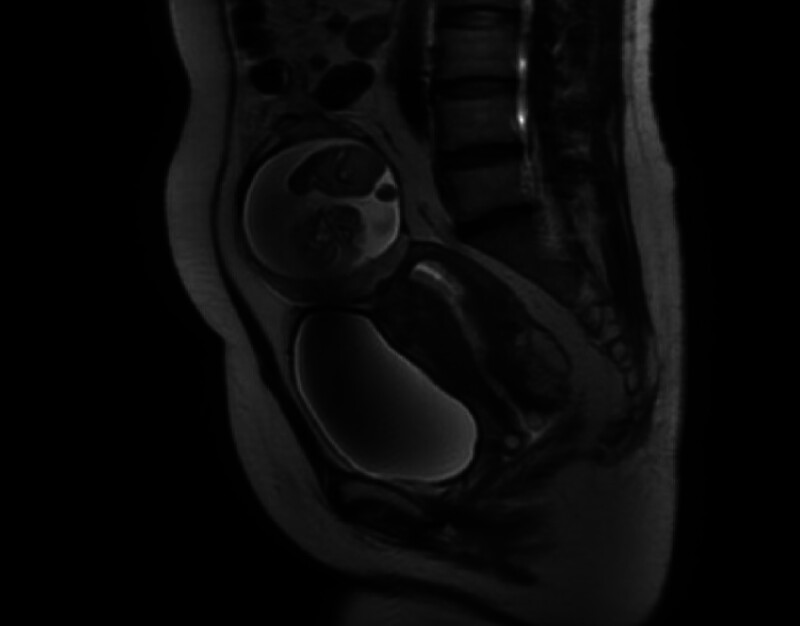
Abdominal pregnancy MRI image. MRI = magnetic resonance imaging.

The enhanced magnetic resonance imaging (MRI) results of the admission examination showed that “a type of oval shaped fetal sac shadow with a size of about 120 × 58 × 86 mm can be seen behind the muscle of the abdominal wall above the bladder in the anterior upper part of the uterus in the abdominal cavity, and fetal imaging can be seen inside. The placental structure can be seen in the lower left anterior part” (Fig. 2). On April 30, 2024, a cesarean section was performed for exploration, intraoperative observation: the longitudinal incision in the middle of the abdomen is about 8 cm in length. After washing hands, exploration revealed the presence of an amniotic sac, which was punctured and cloudy amniotic fluid flowed out of the sac. The amniotic fluid was aspirated, and the stillborn fetus was removed. The fetal length was about 16 cm, and the umbilical cord was interrupted. The diameter of the placenta was about 8 cm. The placenta was attached to the omentum and the left fallopian tube, and the left fallopian tube lost its normal shape. There was a rupture in the ampulla, with a large amount of old blood clots attached. The uterus was of normal size and surface congestion, and inflammatory exudation was observed. A fibroid nodule with a diameter of about 2 cm was palpated on the anterior wall of the uterus, and no obvious abnormalities were observed in the appearance of the left ovary and right adnexa. Laparotomy and left fallopian excision and removal of uterine fibroids and partial omentectomy. Intraoperative bleeding of about 200 mL, without blood transfusion treatment. The patient recovered well 3 days after surgery and was allowed to be discharged.

## 3. Discussion

Abdominal pregnancy includes primary abdominal pregnancy and secondary abdominal pregnancy. The diagnostic criteria for primary abdominal pregnancy still follow the 1942 Studdiford criteria^[2]^: bilateral fallopian tubes and ovaries have a normal appearance; no formation of uterine peritoneal leakage; pregnancy only exists in the abdominal cavity, and there is no possibility of tubal pregnancy. Some studies have found a certain correlation with endometriosis.^[3]^ After careful examination of the medical history, the patient reported experiencing symptoms of amenorrhea. Around 2 months after amenorrhea, the patient experienced sudden lower abdominal pain, which was severe and lasted for about 2 to 3 hours before the symptoms improved. Therefore, it was not taken seriously. Therefore, preoperative consideration suggests that the patient may be a case of secondary abdominal pregnancy. During the operation, the left fallopian tube lost its normal shape, the ampulla was ruptured, and a large number of old blood clots were visible. The placenta is attached to the omentum and the left fallopian tube. The preoperative diagnosis was verified by the exploration results. The choice of treatment for abdominal pregnancy is mainly determined based on gestational age.

Early pregnancy in the abdominal cavity requires careful exploration of the pelvic and abdominal cavities, as the placenta has not yet formed. The surgical scope and method are determined based on the location and size of the gestational sac implantation.^[4]^ The treatment of mid-pregnancy in the abdominal cavity includes conservative and surgical treatments. The key to successful treatment of mid-pregnancy in the abdominal cavity is the handling of the placenta. The risks and complications of surgery vary depending on the location of placenta attachment and the depth of placenta implantation. Therefore, it is crucial to evaluate the site of the gestational sac, the location of placenta attachment, and the main blood supply through preoperative examinations such as ultrasound, computed tomography, or MRI. Conservative treatments for mid-abdominal pregnancy include drugs such as methotrexate (MTX), potassium chloride, and mifepristone,^[5–7]^ combined with selective placental vascular embolization and long-term follow-up observation. However, the drawbacks of conservative treatment are also evident, such as slow regression of the placenta and fetal sac, the possibility of rupture of the gestational sac, placental detachment leading to intra-abdominal bleeding, abdominal infection, and the impact of MTX medication on ovarian function and female fertility. Therefore, surgery is still the most common and effective treatment plan for abdominal pregnancy.^[8–10]^ There are two types of surgeries: laparoscopic surgery and open surgery. Laparoscopic surgery is suitable for patients with early abdominal pregnancy. In this case, the placental attachment site was fully evaluated by ultrasound and enhanced MRI before surgery, Ultrasound shows “placental echo can be seen behind the echo of the rectus abdominis muscle, and the main blood supply comes from the iliac vein.” MRI shows “a type of elliptical fetal sac shadow, about 120 × 58 × 86 mm in size, can be seen behind the muscle of the abdominal wall above the bladder in the anterior upper part of the uterus. Fetal imaging can be seen inside, and placental structure can be seen in the lower left anterior part.” After multiple communications with ultrasound and imaging departments before surgery, the MRI shows that the omentum tissue is wrapping the gestational sac downwards. During the enhancement phase, the main blood vessels supplying the placenta may come from the omentum. It is evaluated that the surgery can completely remove the placenta, so preoperative preparation is actively carried out.

As a rare ectopic pregnancy, although with the improvement of social and economic levels and the popularization of prenatal health knowledge, most cases of abdominal pregnancy can be detected and terminated in the early stages of pregnancy. Therefore, cases of abdominal pregnancy lasting until mid-pregnancy are extremely rare. For this case, sufficient communication should be made with the patient and their family before surgery, and the gestational sac and placental implantation site should be evaluated through ultrasound, MRI, and enhanced computed tomography^[11–13]^ examinations. Other treatment methods, such as placental vascular embolization, should be used to minimize intraoperative bleeding and completely remove the placenta. Of course, there are currently some cases where the placenta is left in the abdominal cavity for natural absorption. The appropriate use of MTX or interventional therapy after surgery can also help reduce bleeding. However, conservative treatment or disposal of the placenta in situ requires more evidence-based medicine.

## Author contributions

**Supervision:** Qi Wu.

**Writing – original draft:** Qi Wu.

**Writing – review & editing:** Suning Bai.

**Funding acquisition:** Lina Han.

**Resources:** Lina Han, Liyun Song.
